# High-Level Modeling and Simulation Tool for Sensor Conditioning Circuit Based on Artificial Neural Networks

**DOI:** 10.3390/s19081814

**Published:** 2019-04-16

**Authors:** Javier Alejandro Martínez-Nieto, Nicolás Medrano-Marqués, María Teresa Sanz-Pascual, Belén Calvo-López

**Affiliations:** 1 Electronics Department, National Institute of Astrophysics, Optics and Electronics (INAOE), Puebla 72840, Mexico; materesa@inaoep.mx; 2 Group of Electronic Design (GDE), University of Zaragoza, 50009 Zaragoza, Spain; nmedrano@unizar.es (N.M.-M.); becalvo@unizar.es (B.C.-L.)

**Keywords:** Artificial Neural Networks, CMOS ASICs, embedded systems, circuit simulation, high-level modeling

## Abstract

For current microelectronic integrated systems, the design methodology involves different steps that end up in the full system simulation by means of electrical and physical models prior to its manufacture. However, the higher the circuit complexity, the more time is required to complete these simulations, jeopardizing the convergence of the numerical methods and, hence, meaning that the reliability of the results are not guaranteed. This paper shows the use of a high-level tool based on Matlab to simulate the operation of an artificial neural network implemented in a mixed analog-digital CMOS process, intended for sensor calibration purposes. The proposed standard tool enables modification of the neural model architecture to adapt its characteristics to those of the electronic system, resulting in accurate behavioral models that predict the complete microelectronic IC system behavior under different operation conditions before its physical implementation with a simple, time-efficient, and reliable solution.

## 1. Introduction

Nowadays, microelectronic integration technologies allow for the fabrication of systems which perform complex signal processing at a relatively low cost, so that they can be embedded in any household device. As a result, and in combination with the rise of new low-power radio frequency (RF) transmission standards, the paradigm of Internet of Things (IoT) has emerged, where devices can interact with each other, transmitting information gathered from themselves or from the environment in order to improve the whole system’s functionality. The information is collected by low-cost sensors that provide a raw electrical signal related in a nonlinear way to the corresponding physical magnitude to be measured. Therefore, analog pre-processing is mandatory before feeding the signal into the digital processing system, which is typically a microcontroller. Furthermore, if the analog preprocessing includes linearization, the resolution requirements for the Analog-to-Digital Converter (ADC) are relaxed.

Programmable analog circuits provide both the capability to linearize the sensor output response and to standardize it by adjusting the conditioning circuit characteristic when operation conditions change due to aging, cross-sensitivity, or sensor replacement [1,2,3,4,5,6,7,8]. The use of analog electronics allows for a reduction of the requirements of the ADC in terms of resolution, because the signal is already linearized after the correction process (note that if the input signal to the ADC is non-linear, a higher resolution is required to digitize the non-linear parts). Besides, the analog system can provide both a linearized analog and a linearized digital output (if an ADC converter is considered at the output of the system), whereas a fully digital system only provides the output in the digital domain.

In particular, Artificial Neural Networks (ANN) constitute the most powerful solution, as they are able to adapt their input–output characteristics without previous knowledge of the particular sensor response [9,10,11,12,13,14,15,16,17]. In this approach, training algorithms are used where sensor input-conditioned output data pairs, which represent the expected input–output characteristic, are iteratively fed to the system, so that the ANN-free parameters (called weights) are adjusted until a maximum permissible error between the expected and the actual output is reached [18,19,20]. The use of registers for weight storage eases the training processes. These mixed-mode solutions take advantage of the low-voltage low-power characteristics of analog processing electronics, as well as the digital programmability of register-based structures, lending great flexibility to the system.

In order to ensure design robustness, it is necessary to analyze its characteristics both in the training and operation modes, under different working conditions (typical, process corners, local mismatch). The really large amount of time required for all these simulations makes them unaffordable at an electrical or physical level by using conventional microelectronic design tools. As a matter of fact, simulations can last for weeks without guaranteeing a convergent solution.

The goal of this work is to come up with a solution for high-level simulation of mixed-mode ANNs. It is based on the toolbox for artificial neural networks provided by Matlab [21], but taking into account the electronic behavior predicted by the circuit simulator. To achieve that, some modifications are applied to the library components, so that local effects in the circuit operation (circuit mismatches, load effects, and so on) can be added. In this way, reliable information of the system behavior is provided in a fraction of the time required by physical or electrical simulators.

It is worth mentioning that similar works were not found in the literature, because they generally focus on presenting the results once the circuit has been implemented at a hardware level. Therefore, this work describes a kind of methodology or tool that can be used to simulate the operation of the ANN-based conditioning circuits in order to provide a software test solution able to simulate the electronic circuit in those processes where the electrical tool oftentimes does not reach a solution, due to the difficulties with the numerical methods of the simulator.

Preliminary results in [22] have allowed for verification of the characteristics of an artificial neural network, CMOS circuit design for sensor linearization under different working conditions. This paper provides more insight into the methodology used to fully customize the Matlab ANN toolbox in order to introduce the electronic models within the ANN architecture. The performance verification of a neural network by correcting offset, gain, non-linearity, and cross-sensitivity errors for different non-linear sensor responses is also presented by making a comparative analysis of three network architectures, thus verifying the tool capability when a different number of both neurons and inputs is considered. Moreover, a study of the effects of mismatch between neurons is also carried out by generating high-level models from Monte Carlo electrical simulations for each building block within the neuron, thus emulating more realistic behaviors of the ANN-based system.

The paper is structured as follows: Section 2 presents the CMOS mixed-mode ANN architecture designed for sensor calibration, and a description of the main building blocks that make up the neural system. Taking into account the difficulty of simulating the full circuit operation with electrical modeling tools, Section 3 presents the proposed customization of the Matlab ANN toolbox, as well as the library modifications required to address these issues and, therefore, ensure convergence with a simple but reliable solution. The validation of the proposed methodology is shown in Section 4, where different training and simulation results are presented by considering the complete neural network system for different sensor calibration cases. The circuit robustness to mismatches is demonstrated by using high-level models for Montecarlo simulations in Section 5, whereas Section 6 shows the system’s capability to operate with more than one input and thus compensate for the temperature effects on the sensor response. Finally, conclusions are drawn in Section 7.

## 2. ANN-Based Microelectronic Circuit for Sensor Conditioning

**Figure 1:**
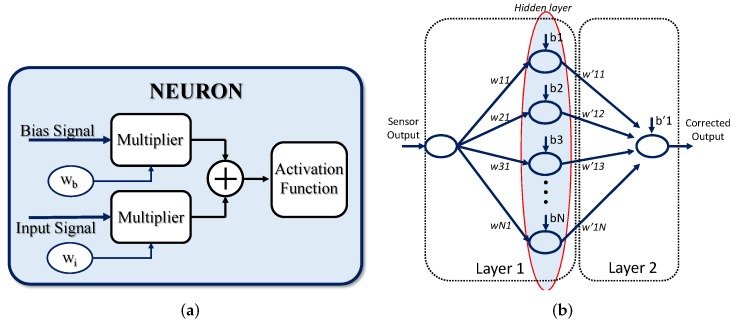
Artificial neural network (ANN) for sensor signal conditioning: (**a**) neuron block diagram, and (**b**) ANN architecture.

The basic processing unit in ANN-based systems is a neuron, whose general structure is shown in Figure 1a. It consists of three main building blocks: a linear analog-digital multiplier, which multiplies either the input or the intermediate-layer signals by a set of digital weights; the addition circuitry, which carries out the sum of two or more weighted signals; and the activation function circuit (AF), which generates the processor unit output by performing a nonlinear operation with the previous weighted sum.

A typical ANN block diagram based on a perceptron processing architecture for sensor signal conditioning is shown in Figure 1b. The system consists of two processing layers: the first one is integrated by the input node and the hidden layer with *N* neurons, and the second by the output layer. The raw signal provided by the sensor is fed to the processing architecture. In the first layer, neurons weight the input data using the coefficients wX1, where *X* ranges from 1 to *N*. An additional signal called bias, with a constant value set to 1, increases the degrees of freedom of the system as it is multiplied by an extra variable coefficient, bX. The sum of both the weighted input and bias signals is also carried out in this layer, and the operation result is processed by a nonlinear operation in order to obtain the output of each neuron in the hidden layer. A weighting of the neuron outputs from the hidden layer is carried out in the last layer, and an addition of the resulting signals with another weighted bias signal is made before providing the system output.

To design a suitable analog sensor conditioning system based on this computing paradigm, a careful choice of the architecture, the processing elements, their connectivity, and the electrical magnitude to be processed is required. Therefore, the sensor output type (current, voltage), level (millivolts, microvolts), and range determines some of the main characteristics of the processing electronics.

### 2.1. Multiplier and Adder

**Figure 2:**
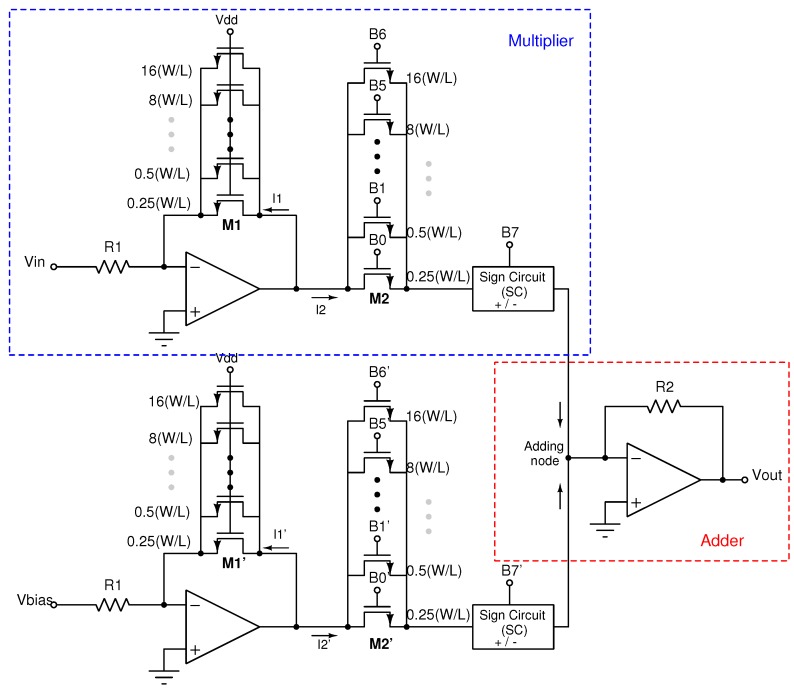
Eight-bit mixed-mode programmable multiplier and adder.

Figure 2 shows the implemented analog-digital multiplier. It is based on a programmable gain amplifier (PGA) and a sign circuit (SC). The multiplier consists of a resistance $R_{1}$, an operational amplifier, and two arrays of PMOS transistors, $M_{1}$ and $M_{2}$, operating in a triode region and, thus, acting as active resistors. $R_{1}$ converts the input voltage $V_{in}$ to a current, and $M_{1}$−$M_{2}$, which work as a linear current divider and set up the gain, weighting $V_{in}$ [23]. The transfer function of the multiplier is given by:

(1) $$I_{2}=\frac{1}{R_{1}}\frac{{\left(W/L\right)}_{2}}{{\left(W/L\right)}_{1}}V_{in}$$

The activation coefficient (weight) is set by turning some of the transistors in the arrays on or off, and thus adjusting the equivalent size ratio (W/L) of $M_{1}$ and $M_{2}$ as well as their equivalent conductances. The maximum gain is 1 because every transistor in parallel forming $M_{1}$ is always on, while the transistors in array $M_{2}$ are controlled by the digital word. Therefore, if maximum gain is desired, all transistors in $M_{2}$ should be on by setting all the bits in the digital word to “high”. Note that the minimum increment between each possible weight is related to the equivalent conductance *g* of the transistor arrays. The digital word determines the total conductance value in $M_{2}$, whereas the conductance in $M_{1}$ is fixed. Because the conductance is defined by:

(2) $$g=\mu_{n}C_{ox}\frac{W}{L}\left(V_{GS}-V_{TH}\right)=k\cdot \frac{W}{L}$$

and, due to the connection of the transistors in the multiplier, the parameter *k* is the same for $M_{1}$ and $M_{2}$; thus, the conductance ratio value only depends on the size ratio (W/L), and therefore, on the number of switched-on parallel transistors.

Each multiplier is designed so that, depending on the 8-bit value stored in a digital register, the output is modulated by the fixed-point weight values, which range from −127 to 127 [24,25,26]. Gain is controlled by a 7-bit digital word ($B_{6}$…$B_{0}$), having 128 possible weight values between 0 and 1. The MSB ($B_{7}$) controls the operation sign, providing weight values from −1 to +1 [27].

**Figure 3:**
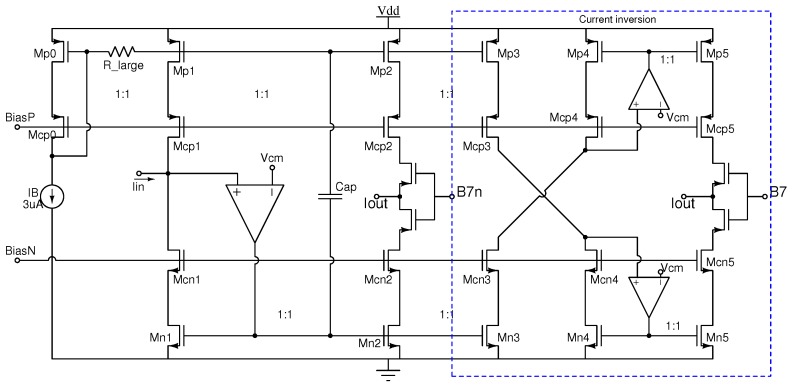
Sign circuit (SC).

The sign circuit at the output of the multiplier determines the direction of the current, thus allowing for negative weights. Figure 3 shows the electronic topology. It is implemented with a dynamic class AB current mirror, providing two output branches: one for the forward current, and the other for the inverted one. The topology is based on the quasi-floating gate approach to achieve a class-AB operation [28,29], thus handling current levels higher than the bias current. In standby, the implementation is equivalent to a conventional class A current mirror formed by transistors $M_{N1}$−$M_{N2}$ and the operational amplifier, and the input and output branches are biased by a DC current $I_{B}$ provided by the current sources $M_{P1}$ and $M_{P2}$. Under dynamic conditions, the configuration achieves class AB operation by transforming transistors $M_{P1}$ and $M_{P2}$ into dynamic current sources. By activating or deactivating the output branches with a control bit, namely the most significant bit (MSB) of the digital word used to set the weight, it is possible to select the current direction. High-precision cascode mirrors were used in order to obtain the highest accuracy in the current copy and, therefore, the highest symmetry between positive and negative weights.

To sum up all the weighted signals, the output of each multiplier was connected at the input of the transimpedance amplifier, which provides a low impedance node for the addition of current signals, and also carries out the current–voltage conversion. In Figure 2 the schematic diagram of a neuron with two weighted signals combined is shown, and the output voltage is defined as follows:

(3) $$V_{out}=\frac{R_{2}}{R_{1}}\cdot \left[\frac{{\left(W/L\right)}_{2}}{{\left(W/L\right)}_{1}}V_{in}+\frac{{\left(W/L\right)}_{2^{\prime }}}{{\left(W/L\right)}_{1^{\prime }}}V_{bias}\right]$$

The transimpedance amplifier consists of an operational amplifier and a feedback resistor $R_{2}$. The operational amplifier configuration consists of a two-stage topology with buffer, which is designed to have a rail-to-rail characteristic because the output layer of the ANN, where all the signals coming from the input layer are added, provides a network output close to the supply voltage rails.

### 2.2. Non-Linear Activation Function Circuit

**Figure 4:**
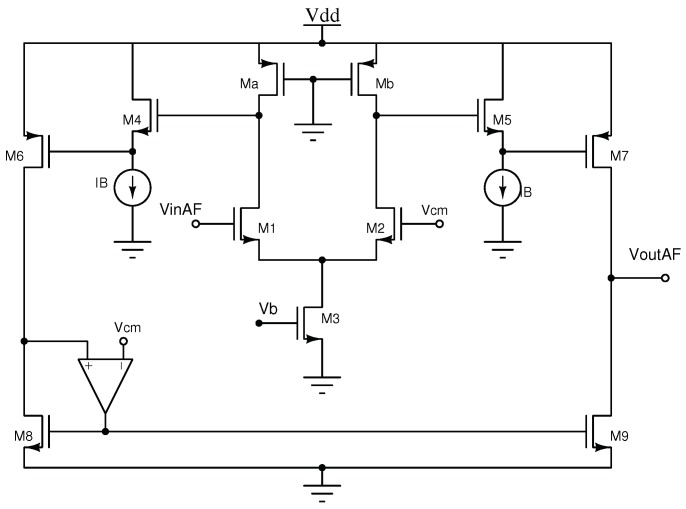
Non-linear activation function circuit.

The non-linear output circuit that generates the activation function is presented in Figure 4. It consists of a differential NMOS input pair with active loads, particularly the transistors $M_{a}$ and $M_{b}$, operating in the linear region. The transistors $M_{4}$−$M_{5}$ act as level shifters, and the differential output is converted into a single output by current mirrors. The amplifier in the high-precision current mirror ($M_{8}-M_{9}$) sets the common mode $V_{cm}=0.9$ V at the output circuit, and is implemented by a simple differential pair.

The electrical characteristic corresponds to a continuous and differentiable logistic function [30,31,32,33,34], thus allowing for the use of a back-propagation training algorithm. It is worth mentioning that the differential pair is the basis of the proposed activation function circuit because of the symmetrical output characteristic, and the well-defined maximum and minimum saturation levels. It also presents a symmetrical response around the origin in order to allow for positive and negative input values, and shows an output node with a well-defined common-mode voltage.

**Figure 5:**
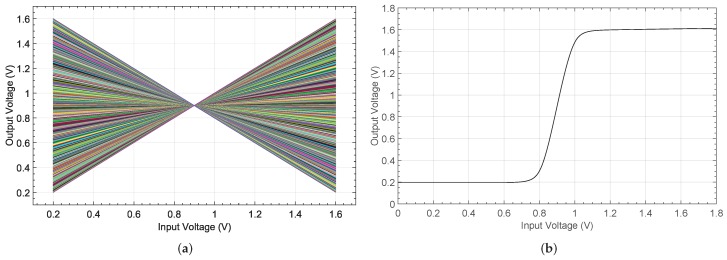
Electrical response for the: (**a**) multiplier and (**b**) activation function circuits.

The electrical response for both the analog-digital multiplier and the activation function circuit is presented in Figure 5a,b, respectively. The simulations were carried out with a UMC 0.18 μm CMOS technology and 1.8 V power supply by using the Cadence tool. For the multiplier circuit, a maximum relative error of 1.6% was obtained when it was compared with an ideal characteristic generated numerically. On other hand, a maximum absolute error of 0.049V was obtained for the non-linear circuit.

## 3. High-Level Simulation Tool for ANN-Based Microelectronic Circuits

Once all the building blocks are designed and individually tested, it is necessary to verify their operation within a complete ANN architecture working as a conditioning circuit. However, training the network to match the correct input-output transfer function is previously required. Due to the complexity of the system and the number of elements composing it, the simulation in the electrical/physical circuit design software package (e.g., Cadence), requires programming in the corresponding high-level tool (e.g., OCEAN [35]). These simulations take up to weeks to be completed, and do not guarantee the convergence of the process.

To speed up the simulation of the network architecture in the training phase, we propose using the ANN toolbox provided by Matlab. A network architecture which incorporates the characteristics of the electronic system must be defined for this purpose. The toolbox allows for selection of different ANN models, arithmetic functions, and network architectures, thus providing high flexibility. However, it is a numerical tool, so the use of electronic circuit models in the network operation is not straightforward. In addition, the ANN architecture is defined by neuron layers in the toolbox. Hence, processors in the same layer use the same multiplier and nonlinear operations, thus limiting the possibility of simulation of mismatch effects or corner analysis. Finally, biases are considered special inputs, set to 1 by default.

### 3.1. Neural Modeling

Several changes have to be carried out in the ANN configuration provided by the toolbox. First, it is necessary to add to the toolbox the mathematical models that represent the operations of the neuron’s electronic building blocks. For this, high-level models of the arithmetics involved in the network are defined from the electrical simulations.

Matlab describes the weight–input interaction at layer level, so that the processors in a layer use the same interaction model, usually the dot product for multilayer perceptron networks. In order to replace the standard product operation for a description closest to the electrical operation, the high-level model of the electronic multiplier implemented must firstly be modeled. For this, the simulation results obtained from the microelectronic design tool under typical operating conditions for the $2^{N}$ different values in the weight register multiplying 100 values of the analog input are applied as input arguments for a multilinear regression model. The resulting model is represented as:

(4) $$V_{out}=c_{1}+c_{2}\cdot V_{in}+c_{3}\cdot w+c_{4}\cdot V_{in}\cdot w$$

where *w* corresponds to the weight value, and $c_{1}$ = 0.9006, $c_{2}$ = $-2.38\times 10^{-5}$, $c_{3}$ = −0.8946, and $c_{4}$ = 0.9930 are coefficients that describe the mathematical model.

**Figure 6:**
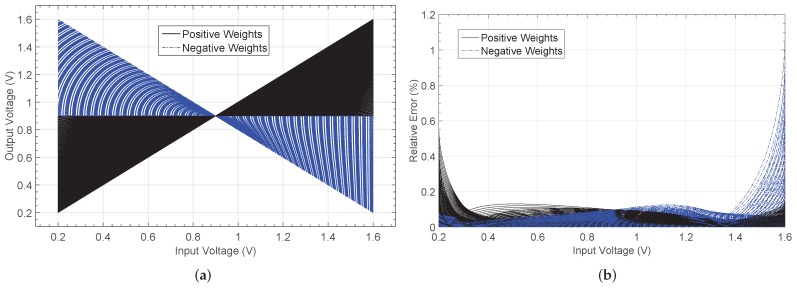
High-level simulated mixed-mode 8-bit multiplier operation: (**a**) model response and (**b**) relative error by comparing with the electrical characteristic.

Figure 6a shows the output characteristic for each activation weight for an input voltage range of 1.6 V, whereas the relative error found by evaluating and comparing the model with the electrical simulations is presented in Figure 6b. Note that the electrical simulations were carried out with a UMC 0.18 μm CMOS technology and 1.8 V power supply by using Spectre as circuit simulator within the Cadence IC design tool. A maximum relative error of 1.04% is obtained when both positive and negative weights are considered.

Similarly, the neural output function is defined as a neural layer property. As in the multiplier modeling, the standard logistic or hyperbolic tangent output function used in the toolbox for hidden layers in an ANN have to be replaced by a realistic behavior of the electronic circuit shown in Figure 4, obtained from the electrical simulator.

Two modeling alternatives can be considered. The first one consists of an approximation based on high-order polynomials:

(5) $$V_{out}=\frac{p_{1}\cdot V_{in}^{5}+p_{2}\cdot V_{in}^{4}+p_{3}\cdot V_{in}^{3}+p_{4}\cdot V_{in}^{2}+p_{5}\cdot V_{in}+p_{6}}{V_{in}^{5}+q_{1}\cdot V_{in}^{4}+q_{2}\cdot V_{in}^{3}+q_{3}\cdot V_{in}^{2}+q_{4}\cdot V_{in}+q_{5}}$$

where $p_{1-6}$ and $q_{1-5}$ refer to the polynomial model coefficients.

**Figure 7:**
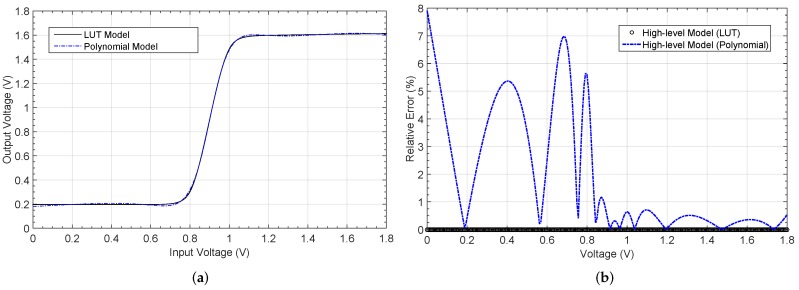
Non-linear logistic function simulated in MATLAB. (**a**) Responses by considering both the high-order polynomial based model and the look-up table, and (**b**) relative error when compared to the electrical characteristic.

A second approach consists in using a look-up-table (LUT) where the stored equispaced values describe the non-linear operation. Figure 7a shows the activation function generated by both the rational model and the LUT (with 200 memory positions). The relative error when comparing both responses with the electrical simulation is presented in Figure 7b. A maximum value of $e_{r}=7.8\%$ is appreciated when the polynomial model is evaluated, whereas a null error is obtained for the LUT case. According to the results, LUT-based nonlinear function modelling is more appropriate due to its simplicity and higher accuracy, and this approach also provides more accurate function saturation levels compared to the high-order polynomial model, as well as how it requires lower computing time.

**Figure 8:**
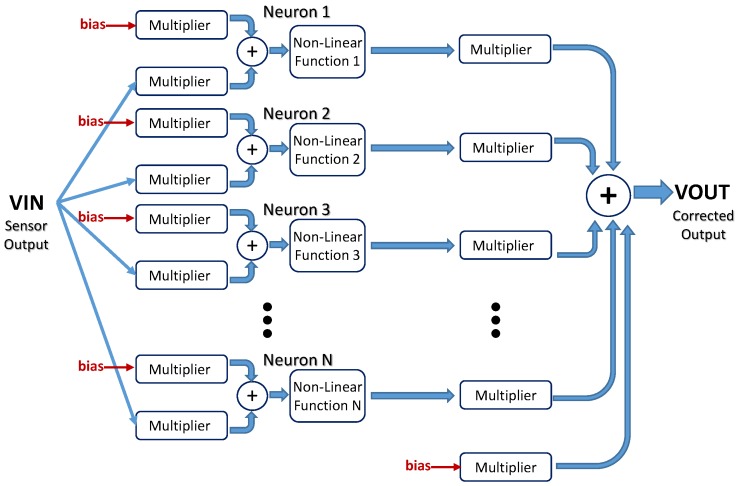
Detailed block diagram of the ANN.

A detailed block diagram of the electronic circuitry required to implement the ANN architecture is shown in Figure 8. Each processor in the first layer consists of two multipliers whose inputs are the sensor signal and the bias input. The outputs of both multipliers are added and fed to the nonlinear function circuit. In the output layer, the multipliers weight both the outputs from the previous layer and the bias input. The output signal results from the addition of the outputs of all these multipliers.

The ANN toolbox is used to define the neural network implementation, and for the training and simulation processes. When a network structure is defined with the tool, it is possible to select different network architectures, but the arithmetic functions within the neuron and the interconnections between the neuron layers are always predefined by the tool.

In order to refer to the designed multiplier block, it was necessary to change the basic product function by the numerical high-level model presented above. Note that the derivatives of the function, with respect to both the weight and input, were also required to be used in the training process. For the logistic function, the procedure was very similar. The predefined non-linear function was changed by the mathematical model defined by the LUT, where its derivative was also included. Because it consists of a table, it was simply programmed to be able to address the appropriate position depending on the input voltage value. Finally, for the addition operation, the sum operator was modified to take into account the circuit common mode voltage of 0.9 V.

Finally, the Matlab toolbox considers neuron biases as special inputs with a constant value equals to “1” that is multiplied by its corresponding adjustable weights. In a voltage-mode analog processor, the bias input must be tied to the maximum processing voltage. In our case, the maximum input voltage was limited to 1.6 V [36]. This means the neural model in the toolbox has to be modified by discarding the bias connections in the architecture and including an additional weighted input to the network at a constant 1.6 V value tied to all the processors in the neural architecture, thus emulating the neural biases in the electronic circuitry.

On the other hand, because the toolbox assigns identical characteristics to the processors in a neural layer, an analysis of mismatch or loading effects in the proposed electronic architecture is not direct at high-level simulation. To allow different processors to behave differently, therefore enabling a reproduction of the effects of non-idealities caused by mismatch, the components which behavior is analyzed must be defined in separate layers, modifying the input, bias, and layer connection parameters available in the structure provided by Matlab to define the neural model. These parameters are arranged in matrices composed by zeros and ones, where a position (x,y) in the layer connection matrix defines the presence (1) or absence (0) of connection from layer *y* to layer *x* (in a similar way as for bias and input connections).

**Figure 9:**
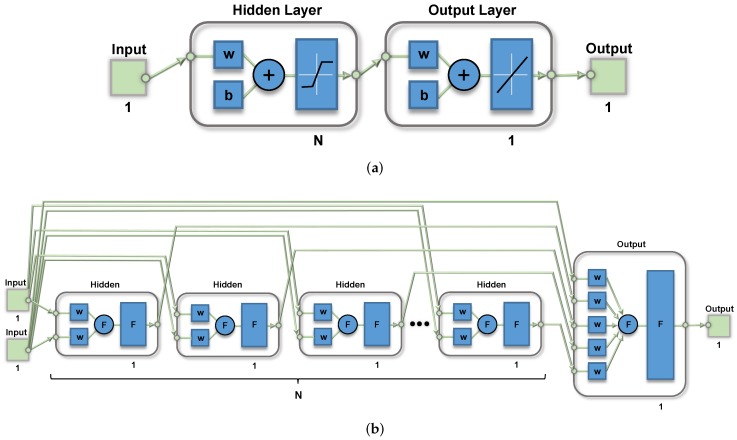
(**a**) ANN defined by Matlab with *N* processors in a single layer and (**b**) modified ANN definition with each processor in an independent layer.

Figure 9 shows the architecture of a neural network consisting of *N*-processors in a hidden layer defined as a (a) single N-processors layer, or (b) *N* different single-processor layers, properly interconnected to the input and output nodes, thus allowing different behaviors for each processor. With this change in the neural definition, it is possible to set different models for both the multiplier and logistic circuits in each layer, thus emulating more realistic behaviors of the whole system. Figure 9b shows this processor arrangement, as well as an additional network input acting as a bias voltage to permit how its value equals to 1.6 V, instead of the default value 1. Note that two multiplier inputs are added in each neuron in the hidden layer, and (N+1) in the output layer because of the additional bias input.

### 3.2. Learning Constraints

In multilayer perceptron networks, the backpropagation (BP) learning algorithm is used [37]: Input data is fed forward through the network to optimize the weights between neurons, where its adjustment is done by backward propagation of the error during the training phase. The network takes the input and target values in the training data set and changes the value of the weighted links to reduce the difference between the output and target values. The error is minimized across many training cycles called epochs.

The use of an efficient training algorithm to find the proper weight values presents some issues, so that it requires several modifications in order to be useful when considering the restrictions related to the operator mathematical models and the weight discretization. In the electronic implementation, weight values are stored in an 8-bit digital register and are limited to a ±1 range, so the learning algorithm must also include these constraints in the possible weight values. To do so, the learning technique used, which is based on the powerful *Levenberg-Marquardt* algorithm [38,39,40], was modified by limiting the weight range to ±1 and carrying out an 8-bit discretization at each learning iteration. In addition, both the product and non-linear function derivatives, required in most of these algorithms, were fitted to those corresponding to the electronics high-level models used in the neural network definition. Note that although it is possible to use other algorithms, the selected one is the fastest and most efficient for ANNs in function approximations tasks [21]. Therefore, the training network function “trainlm” was defined within the toolkit in order to update the weight and bias values according to this optimization, which is a supervised algorithm based on the mathematical quasi-Newton method [41]. The most important training parameters used in the learning phase are summarized in Table 1.

## 4. Sensor Conditioning

The neural network CMOS system designed for sensor conditioning shown in Figure 8 was considered in order to validate the robustness of the modified high-level tool described above.

Three non-linear characteristics are usually considered in order to train and simulate the network structure. These sensor responses (thermistor, hall-effect, and LDR) are experimental data provided by the Aragon Institute of Engineering Research, University of Zaragoza (Spain). Each dataset consists of 120 input–output patterns randomly selected, considering 70% for the training, 15% for test, and the remaining 15% for validation purposes. The target or expected response corresponds with a linear shape with different gains and offsets in order to validate the neural network capability.

The architectures 1-4-1, 1-5-1, and 1-6-1 are defined in order to compare the system performance, taking into account the number of processing elements or neurons constituting the hidden layer. The non-linearity, as well as the offset and gain corrections, are considered and a comparative analysis to determine trade-offs in terms of complexity, correction capability, and estimated power consumption is made, so as to select the best-suited architecture.

### 4.1. Thermistor Placed on a Resistive Divider (NL1)

**Figure 10:**
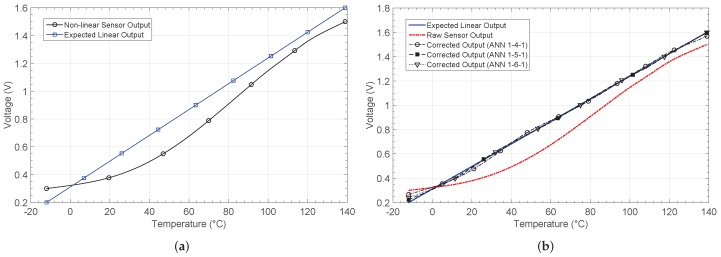
Thermistor output characteristic NL1 (**a**) before and (**b**) after correction.

**Figure 11:**
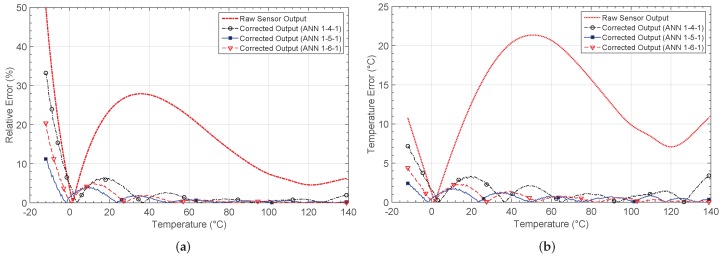
Calculated errors by considering the non-linear characteristic 1 (NL1): (**a**) relative error $e_{r}$ and (**b**) temperature estimation error $e_{T}$.

The first non-linear characteristic results from placing an NTC thermistor on a resistive divider. The output voltage is shown in Figure 10. The linear and expected output are also shown. After the learning phase, the output for each network architecture is shown in Figure 10b. Figure 11a shows the relative error when comparing the responses with the target output, whereas the absolute error in the estimation of the temperature value is shown in Figure 11b.

The network architecture 1-4-1 achieves the solution after 38 epochs or iterations, with a mean square error (MSE) of $3.32\times 10^{-4}$. The other considered architectures more than halve the error, but the 1-6-1 requires 62 iterations to reach the solution. The training for the 1-5-1 architecture was the fastest, with only 32 epochs. Note that the number of iterations shown is the mean value obtained after successfully training the network five times.

Figure 11 shows the relative error, as well as the absolute error in the estimation of temperature before and after calibration. Before calibration, a relative error above 10% exists in most of the temperature range considered. The error decreases considerably after calibration, as shown in Figure 11a, with values lower than 2%.

Figure 11b shows the error when estimating the temperature from the output of the sensor. After calibration with the 1-4-1 network, a maximum error $e_{T_{max}}=7.16$ °C is obtained, whereas for the 1-5-1 and 1-6-1 architectures, the error decreases down to 2.42 °C and 4.39 °C, respectively. The mean error, the number of iterations and the MSE in performance are also presented in Table 2. As the results were obtained from simulations in Matlab, the power consumption was estimated considering the maximum power consumption for all the analog blocks making up each architecture [27,36].

### 4.2. Hall-Effect Sensor to Measure Distance (NL2)

**Figure 12:**
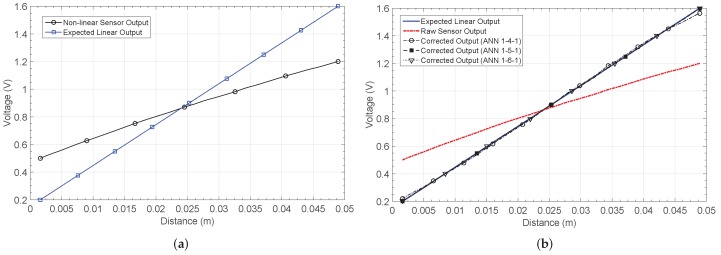
Hall-effect sensor response NL2 (**a**) before and (**b**) after correction.

Another output characteristic considered is the response of a Hall sensor used to measure the position of a permanent magnet. Figure 12 shows the output voltage versus the distance from the sensor to the magnet. The Hall sensor response is more linear than the previous cases, but offset and gain corrections are required.

The output characteristics after calibration are shown in Figure 12b. The solution was reached after 14 epochs for the 1-4-1 network, 19 for the 1-5-1 network, and 18 for the 1-6-1 network. The worst MSE was obtained for the network architecture with less neurons in the hidden layer, just as in the previous cases.

**Figure 13:**
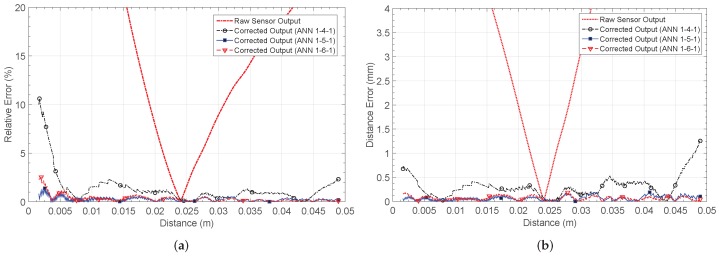
Calculated errors by considering the non-linear characteristic 2 (NL2): (**a**) relative error $e_{r}$ and (**b**) distance estimation error $e_{D}$.

Figure 13a shows the relative error. Before calibration, a maximum relative error of 150% is found. Note that the error is calculated with respect to the target characteristic, which was chosen so as to maximize the output voltage range. For the 1-4-1 network, this error decreases to 10.6%, but the smallest values are obtained for the 1-5-1 and 1-6-1 architectures, with maximum relative errors of 1.39% and 2.43%, respectively. Table 3 summarizes the main characteristics of each network structure.

### 4.3. LDR Placed on a Resistive Divider (NL3)

**Figure 14:**
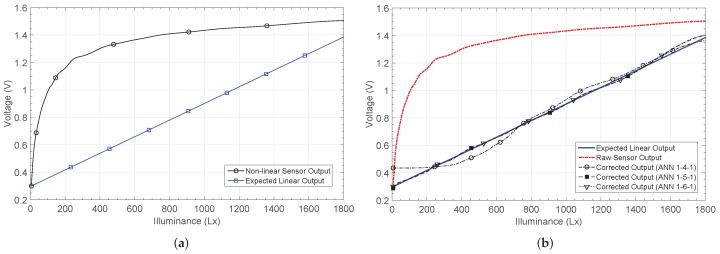
LDR sensor output response NL3 (**a**) before and (**b**) after correction.

An LDR is a variable resistance that changes with the light intensity. When the sensor is placed in a resistive divider, the output voltage shown in Figure 14 is obtained. It is a logarithmic characteristic, and therefore shows the highest non-linearity from all the considered sensors. The X axis corresponds to the illuminance, which is defined as the total amount of light energy reaching an illuminated surface per unit area. In other words, it defines how much the incident light illuminates the surface, and is measured in lux (Lx).

The outputs after calibration are shown in Figure 14b, together with the target characteristic. The 1-4-1 network shows the worst performance in most of the input range, with a maximum relative error after calibration of 45.5%, versus the 80% relative error for the raw sensor.

**Figure 15:**
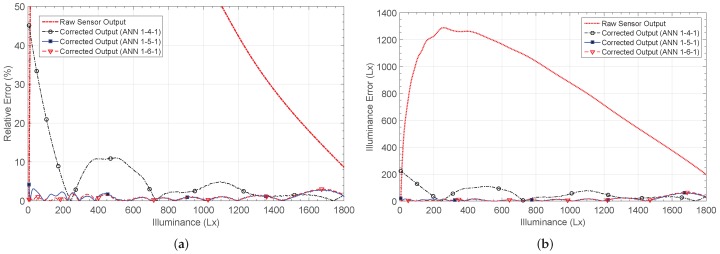
Calculated errors by considering the non-linear characteristic 3 (NL3): (**a**) relative error $e_{r}$ and (**b**) illuminance estimation error $e_{L}$.

The error decreases down to 5% for the 1-5-1 and 1-6-1 networks, as shown in Figure 15a. The minimum error when estimating the illuminance was also obtained using five and six neurons in the hidden layer, and was approximately 65Lx. For the 1-4-1 structure, this error increases to 223Lx. Figure 15b shows the error in the illuminance estimation for the considered architectures. Finally, Table 4 presents their main characteristics.

Based on the previous results, the number of neurons conforming the hidden layer affects the network performance, so that a design trade-off between the number of neurons in the hidden layer and both the network performance and the estimated power consumption exits. In all the considered cases, the 1-4-1 network architecture showed the worst results.

When the network performance is compared for the 1-5-1 and 1-6-1 structures, the difference is negligible most of time. However, the 1-5-1 architecture showed better results in correcting the sensor characteristics. Therefore, it is not worth increasing the number of processing elements—and thus the overall power consumption—if the performance improvement is almost null.

Finally, it can be concluded that the small network architectures considered are capable of conditioning several types of sensor non-idealities by linearizing their output characteristics, in addition to correcting offset and gain errors. So, it would be possible to integrate a single neural network to compensate for different low-cost analog sensors.

## 5. Model Validation

**Figure 16:**
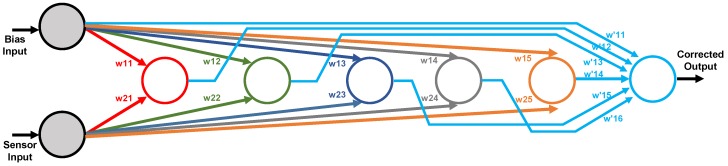
Weight definition of the neural network equivalent architecture.

In order to validate the proper performance of the high-level models extracted in the previous sections, an electrical simulation has been carried out by taking into account the digital weights obtained in the neural network learning phase made with the Matlab tool. The 1-5-1 network architecture was implemented in the electronic design tool by using a conventional 0.18μm CMOS technology with 1.8 V power supply, and the non-linear thermistor response (NL1) was considered for the test. In this way, 16 8-bit digital weights were required to weight the multipliers in each neuron, and Table 5 summarizes the value of each weight with its corresponding digital word. Note that Figure 16 shows a block diagram of the equivalent architecture where different layers are represented with different colors, and their corresponding weight labels are also shown.

**Figure 17:**
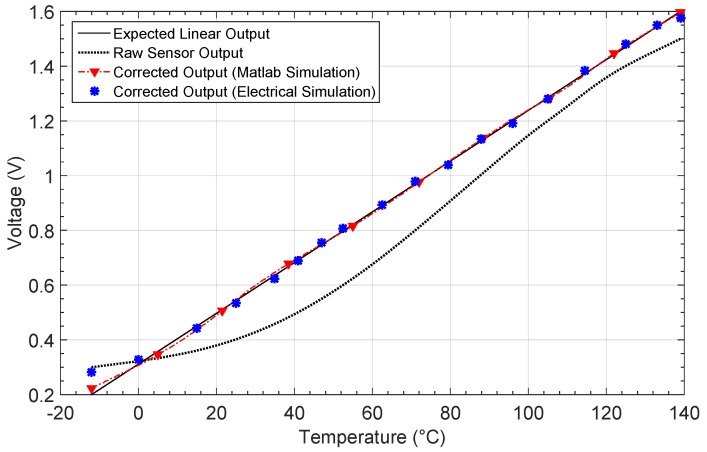
Non-linear output characteristic NL1 compared with the output obtained after electrical simulation.

Note that it was required to divide the electrical simulation process in order to avoid convergence problems, as well as decrease the simulation time. First, the neurons in the hidden layer were processed, so that their resulting outputs were considered as inputs for the output-layer neuron in a second simulation. It is worth mentioning that 18 input values along the non-linear characteristic voltage were considered for the simulation, and the obtained response is shown in Figure 17 where the Matlab output response is also presented for comparison purposes.

**Figure 18:**
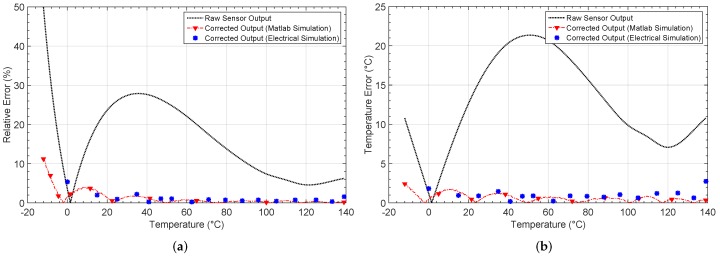
Comparison between the non-linear characteristic NL1 and the corrected output electrical characteristic: (**a**) relative error $e_{r}$ and (**b**) temperature estimation error $e_{T}$.

Figure 18 presents both the relative error and the error in the temperature estimation by taking into account the electrical simulation. Results show a maximum relative error of 5.36%, which is almost 10 times less than the uncorrected response error. Moreover, this error remains below 2%, with most of the temperature span showing congruence with the Matlab simulation. On the other hand, for the temperature estimation error, the maximum value obtained is 2.72 °C with a mean value of 1.02 °C, which is a remarkable difference with the uncorrected characteristic, where a maximum error of 21.35 °C is appreciated. Again, the results were as expected, according to the Matlab high level simulations presented above. Table 6 summarizes the calculated errors when comparing the Matlab and electrical simulation responses with the non-linear uncorrected characteristic.

## 6. Effects of Mismatch

To study the effects of mismatch on the ANN-based system, corner process and Monte Carlo statistical variations were considered for each of the electronic building blocks conforming the neuron. In this way, the required electrical simulations were carried out individually for both the mixed-mode multiplier and the activation function circuits in order to obtain a set of mathematical high-level models for each one. From this data set, six mismatch cases were considered to perform high-level simulations of the whole ANN-based circuit, considering the previously studied practical case of correcting the thermistor sensor output response (NL1).

**Figure 19:**
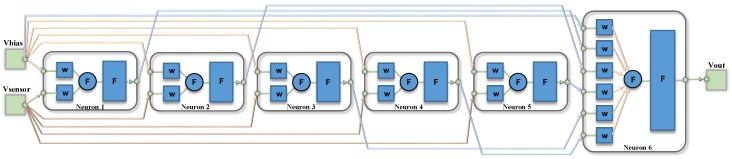
1-5-1 network Matlab definition for studying the effect of mismatch between neurons.

From the performance results presented above, the 1-5-1 architecture was chosen for studying the mismatch effects, and Figure 19 shows the resulting Matlab definition with each neuron interconnected in different layers.

**Figure 20:**
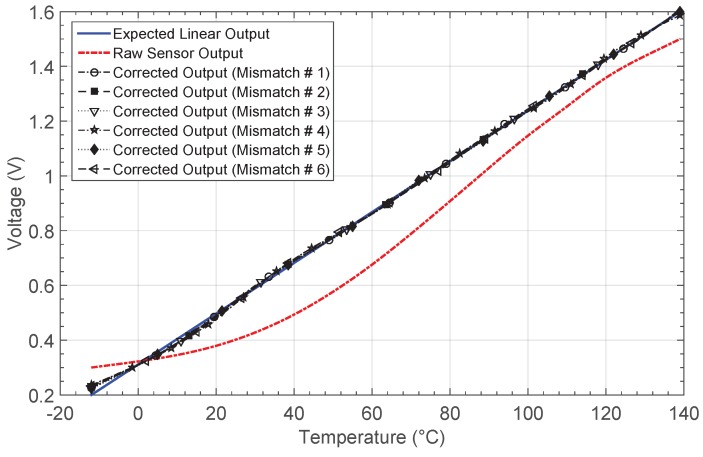
Thermistor output characteristic NL1 before and after correction by considering six mismatch cases between neurons.

**Figure 21:**
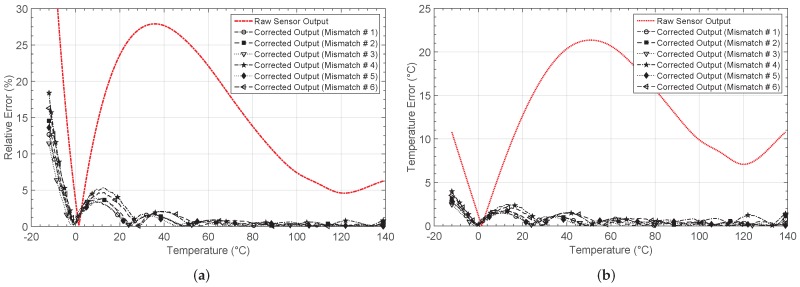
(**a**) Relative error $e_{r}$ and (**b**) error in the temperature estimation $e_{T}$ obtained when mismatch between neurons is considered.

Figure 20 shows the output for each mismatch case after the learning phase, whereas the obtained relative errors and the errors in the temperature estimation are presented in Figure 21a,b, respectively. By considering all the cases, the network system solves the problem after a mean value of 46 iterations. Note that, before calibration, a relative error of higher than 10% is appreciated in most of the temperature range considered, and it decreases after calibration for all the mismatch cases to values lower than 3%. Table 7 summarizes the obtained errors, and the number of epochs after the network training. Note that the mean values are also presented.

The mean value of the maximum error in the temperature estimation remains below 3.12 °C after correction, which is compared with the value of 21.35 °C obtained for the sensor response without correction. Therefore, it can be concluded that although mismatch occurs within the electronic blocks conforming the neuron, the proper system performance is not affected.

## 7. Temperature Cross-Sensitivity Correction

The cross-sensitivity refers to the sensor response variation due to changes in variables other than the main one—that is, the sensor output is not only sensitive to the input signal to be measured, but also to other parameters. In particular, temperature is the physical variable which most affects the performance of a sensor, so that a response correction is necessary to compensate for its effect [42,43,44,45,46]. The goal is to keep the sensor response independent of temperature variations. To do so, a second neuron input is used to provide information of the ambient temperature.

**Figure 22:**
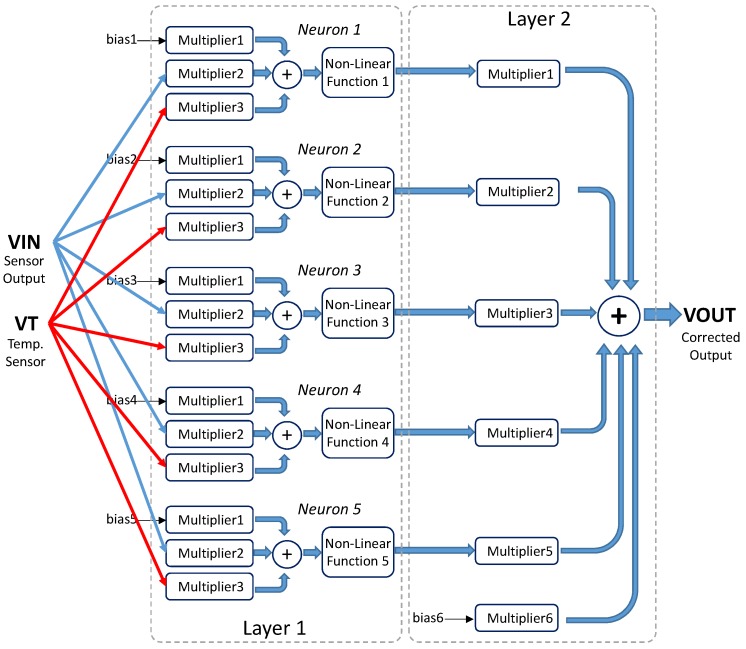
ANN architecture used to correct temperature cross-sensitivity.

The 1-5-1 network architecture was modified in order to add a second input and thus compensate for the temperature effects on the sensor response, as shown in Figure 22. Each neuron in the hidden layer now consists of three multipliers, meaning that the high-level models for these multipliers were changed. Note that the activation function circuit remains the same, as well as the multipliers in the output layer.

**Figure 23:**
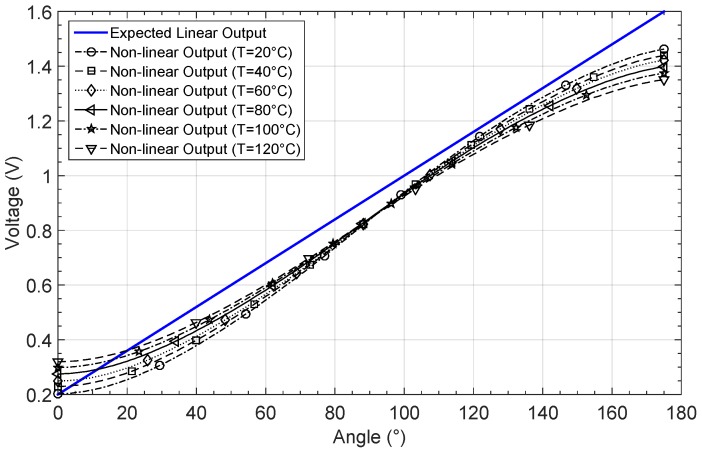
Non-linear giant magneto-resistance (GMR) response by considering different temperatures.

In order to train and simulate the defined network structure, a giant magneto-resistance (GMR) sensor response was considered in order to measure the angular position. The GMR sensor presents a non-linear response, similar to a sinusoidal characteristic. Figure 23 shows the output response by considering an angle input range between 0° and 175°, for six different temperatures. Note that the offset, as well as the gain response, changes with temperature.

**Figure 24:**
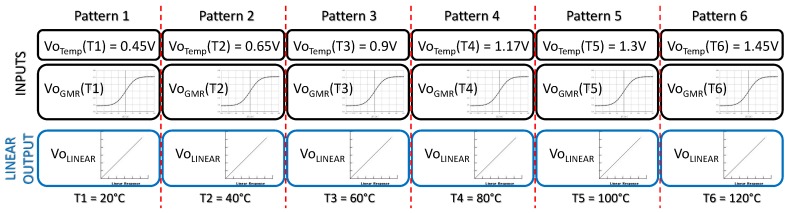
Defined data structure for the learning phase.

Six input–output patterns were used in the learning phase, so the data structure shown in Figure 24 was built. A thermistor was used to measure the temperature, which is also related to a 120 data set corresponding to the non-linear GMR output, as also shown in Figure 24.

**Figure 25:**
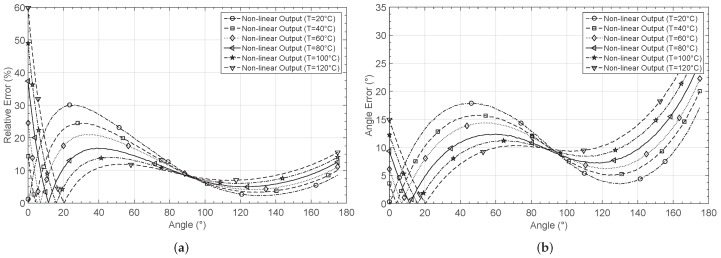
Calculated errors by considering the GMR non-linear characteristic at different temperatures: (**a**) relative error $e_{r}$ and (**b**) angle error estimation $e_{∡}$.

Before correction, both the relative error and the angle-measurement error were calculated at each temperature. Table 8 summarizes these errors, and Figure 25a shows the relative error for each output response when compared with the expected one. The maximum vale is $e_{r}=59.82\%$, and it corresponds to the 120 °C temperature.

Figure 25b shows the error in angle estimation from the six temperature-dependent GMR characteristics before calibration. The mean error is $e_{∡}=10$° and the maximum error is $e_{∡}=31$°, obtained at 120 °C.

**Figure 26:**
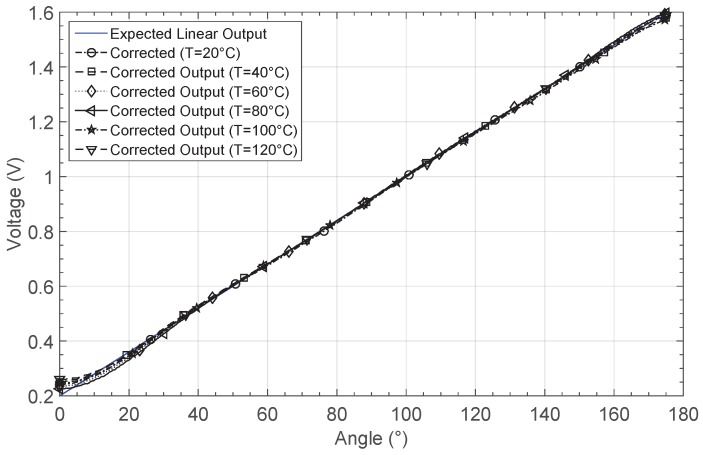
Corrected GMR response by considering different temperatures.

Once the system was trained with the data structure presented before, the corrected outputs were presented in Figure 26. The learning phase requires 83 iterations to solve the problem. Therefore, in order to correct changes with temperature, the learning phase requires more time due the size of the dataset, as well as more processing capabilities.

**Figure 27:**
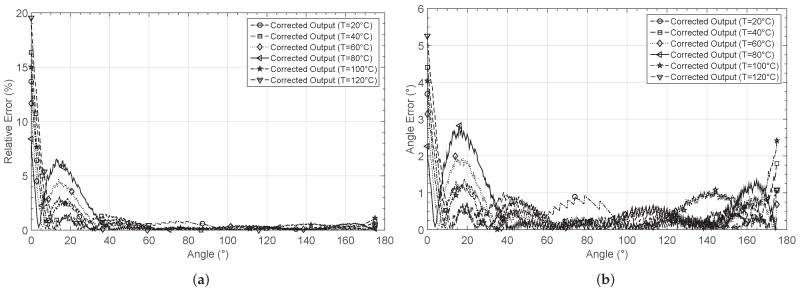
Calculated errors by considering the corrected GMR characteristic at different temperatures: (**a**) relative error $e_{r}$ and (**b**) error in the angle estimation $e_{∡}$.

When comparing with the non-corrected responses, the ANN corrects offset and gain non-linearity errors. Figure 27a shows the relative error for each case when they are compared to the linear target. After correction, the relative errors remain below 2% for most of the input range, particularly within an angle range between 30° and 175°. The relative error increases when the output voltage is around 0.2 V. However, for this output voltage section, the error in the estimation of the angle $e_{∡}$ is in the order of 5°, which is three times smaller than the uncalibrated responses. Figure 27b presents the estimated angle error for the six temperatures. It remains below 2° for most of the input range for all the curves, and below 1° for an input span of 120°. Table 9 summarizes the errors at each temperature for the six corrected outputs.

## 8. Conclusions

This work presented the methodology required to develop a high-level tool based on Matlab to verify the mixed-mode CMOS artificial neural network proper performance designed for sensor conditioning. The modified tool allows verification of the system operation under typical conditions, as well as by considering process variations on each of the building blocks conforming the neuron. Moreover, the proposed modified tool was also verified by considering ANNs with a different number of neurons, weights, and inputs by modifying the connections matrix in the network model provided by the toolbox.

The offset, gain, and non-linearity corrections were considered for different sensors. Results show that the 1-5-1 architecture is the best solution by taking into account the trade-off between performance and power consumption. Moreover, a validation of the model was carried out by simulating the trained neural system at an electrical level in Cadence, and results show that the errors obtained are of the same order as those obtained from the Matlab simulation.

To evaluate the robustness of the whole system, each of the building blocks conforming the neuron were modeled by taking into account process corners and mismatch, and results show that the system is able to reach a successful solution even when the individual operation models within a neuron are different.

Finally, the system capability operating with multiple inputs was verified by adding a second input in order to compensate for the temperature effects on the response of a giant magneto-resistive sensor. Results showed that with input–output pattern sets from the proposed sensor at six different temperatures, the network is able to reach a solution, and a maximum error of 1° in the estimation of the angle is obtained for most of the considered input range.

These results show the utility of the proposed tool to simulate the operation of an ANN-based conditioning circuit, thus providing a reliable solution for mixed-mode ANNs high-level simulations in a period of time less than that required by the microelectronic design tool, which oftentimes does not reach a convergent solution.

## Figures and Tables

**Table 1:** Training parameters for the *trainlm* function defined in the Matlab toolkit.

Parameter	Value	Toolkit Definition
Maximum number of epochs	1000	net.trainParam.epochs
Performance goal	0	net.trainParam.goal
Maximum validation failures	10	net.trainParam.max_fail
Minimum performance gradient	1 $\times \;10^{-7}$	net.trainParam.min_grad
Initial adaptive value *mu*	0.001	net.trainParam.mu
*mu* decrease factor	0.0001	net.trainParam.mu_dec
*mu* increase factor	2	net.trainParam.mu_inc
Maximum *mu*	1 $\times \;10^{15}$	net.trainParam.mu_max

**Table 2:** Comparative summary of the three ANN structures for the non-linear characteristic *NL1*.

Structure	Max. Power (mW)		$e_{T_{\max}}$	$e_{T_{\mathrm{mean}}}$	$e_{r_{\max}}$	$e_{r_{\mathrm{mean}}}$	Epochs	Performance
	Static	Dynamic	(°C)	(°C)	(%)	(%)		MSE
Uncorrected	—	—	21.35	12.64	50	15.73	—	—
1-4-1	0.92	2.1	7.16	1.54	33.22	2.92	38	$3.32\times 10^{-4}$
1-5-1	1.2	2.6	2.42	0.64	11.22	1.16	32	$5.12\times 10^{-5}$
1-6-1	1.5	3.3	4.39	0.75	20.34	1.62	62	$9.22\times 10^{-5}$

$e_{T}$→ Error in the estimation of temperature. $e_{r}$→ Relative error.

**Table 3:** Comparative summary of the three ANN structures for the non-linear characteristic *NL2*.

Structure	Max. Power (mW)		$e_{D_{\max}}$	$e_{D_{\mathrm{mean}}}$	$e_{r_{\max}}$	$e_{r_{\mathrm{mean}}}$	Epochs	Performance
	Static	Dynamic	(m)	(m)	(%)	(%)		MSE
Uncorrected	—	—	$13.5\times 10^{-3}$	$6.0\times 10^{-3}$	150	22.78	—	—
1-4-1	0.92	2.1	$1.3\times 10^{-3}$	$3.0\times 10^{-4}$	10.60	1.32	14	$1.21\times 10^{-4}$
1-5-1	1.2	2.6	$1.9\times 10^{-4}$	$6.6\times 10^{-5}$	1.39	0.25	19	$5.44\times 10^{-6}$
1-6-1	1.5	3.3	$1.8\times 10^{-4}$	$6.6\times 10^{-5}$	2.43	0.31	18	$5.40\times 10^{-6}$

$e_{D}$→ Error in the estimation of distance. $e_{r}$→ Relative error.

**Table 4:** Comparative summary of the three ANN structures for the non-linear characteristic *NL3*.

Structure	Max. Power (mW)		$e_{L_{\max}}$	$e_{L_{\mathrm{mean}}}$	$e_{r_{\max}}$	$e_{r_{\mathrm{mean}}}$	Epochs	Performance
	Static	Dynamic	(Lx)	(Lx)	(%)	(%)		MSE
Uncorrected	—	—	1280	849.05	183.98	80.12	—	—
1-4-1	0.92	2.1	223.75	55.15	45.11	5.86	28	$2.3\times 10^{-3}$
1-5-1	1.2	2.6	62.45	15.54	4.18	1.06	35	$4.54\times 10^{-4}$
1-6-1	1.5	3.3	65.54	15.46	3.01	0.95	24	$4.60\times 10^{-4}$

$e_{L}$→ Error in the illuminance estimation. $e_{r}$→ Relative error.

**Table 5:** Digital weights obtained after the learning phase.

Weight (*w*)	Value	Digital	Weight (*w*)	Value	Digital	Weight (*w*)	Value	Digital
		Word			Word			Word
$w_{11}$	0.2161	00011011	$w_{21}$	0.9885	01111110	$w_{11}^{\prime \prime }$	−0.4917	10111110
$w_{12}$	0.7948	01100101	$w_{22}$	0.5428	01000101	$w_{12}^{\prime \prime }$	0.2279	00011101
$w_{13}$	−0.8041	11100110	$w_{23}$	0.3843	00110001	$w_{13}^{\prime \prime }$	0.7918	01011000
$w_{14}$	−1.0	11111111	$w_{24}$	0.8834	01110000	$w_{14}^{\prime \prime }$	0.6895	01010110
$w_{15}$	−0.1559	10010100	$w_{25}$	−0.1551	10010100	$w_{15}^{\prime \prime }$	0.4113	00110100
–	–	–	–	–	–	$w_{16}^{\prime \prime }$	−1.0	11111111

**Table 6:** Comparison between the Matlab and electrical simulations performed in Cadence by considering the 1-5-1 neural architecture and the non-linear thermistor response (NL1).

Simulation	$e_{T_{\max}}$	$e_{T_{\mathrm{mean}}}$	$e_{r_{\max}}$	$e_{r_{\mathrm{mean}}}$
	(°C)	(°C)	(%)	(%)
Uncorrected	21.35	12.64	50	15.73
Matlab	2.42	0.64	3.94 *	1.16
Electrical	2.72	1.02	5.36 *	1.19

Span of 140 °C. $e_{T}$—Temperature error. $e_{r}$—Relative error.

**Table 7:** Calculated errors considering six mismatch cases between neurons.

Simulation	Relative Error $e_{r}$		Temperature Error $e_{T}$		Epochs	MSE
	Max. (%)	Mean (%)	Max. (°C)	Mean (°C)		
Mismatch Case #1	12.66	1.16	2.73	0.62	70	$5.75\times 10^{-5}$
Mismatch Case #2	14.54	1.25	3.13	0.63	43	$5.92\times 10^{-5}$
Mismatch Case #3	11.45	1.08	2.47	0.54	34	$4.84\times 10^{-5}$
Mismatch Case #4	18.39	1.72	3.96	0.94	25	$12.0\times 10^{-5}$
Mismatch Case #5	13.60	1.07	2.93	0.52	65	$4.08\times 10^{-5}$
Mismatch Case #6	16.27	1.52	3.51	0.75	41	$9.25\times 10^{-5}$
Mean Results	14.48	1.30	3.12	0.66	46	$6.97\times 10^{-5}$

**Table 8:** Calculated errors by considering the GMR non-linear characteristic at different temperatures.

Temperature	Relative Error $e_{r}$		Angle Error $e_{∡}$	
	Max. (%)	Mean (%)	Max. (°)	Mean (°)
20 °C	30.09	12.87	17.87	10.45
40 °C	24.55	11.47	20.02	10.21
60 °C	24.54	10.86	22.26	10.24
80 °C	37.39	10.21	25.22	10.14
100 °C	48.98	10.37	28.21	10.60
120 °C	59.82	10.86	31.08	11.20

**Table 9:** Calculated errors by considering the corrected GMR characteristic at different temperatures.

Temperature	Relative Error $e_{r}$		Angle Error $e_{∡}$	
	Max. (%)	Mean (%)	Max. (°)	Mean (°)
20 °C	13.68	0.69	3.68	0.42
40 °C	16.36	0.74	4.40	0.45
60 °C	11.66	0.77	3.14	0.48
80 °C	8.40	0.96	2.82	0.62
100 °C	15.01	0.79	4.04	0.57
120 °C	19.53	0.80	5.26	0.43
